# Improving the Throughput and Specificity for Small-Molecule Analysis During First-Tier Mass Spectrometry–Based Newborn Screening

**DOI:** 10.3390/metabo16070443

**Published:** 2026-06-25

**Authors:** Samantha L. Isenberg, Charles A. Pickens, Rachel Lee, Carla Cuthbert, Konstantinos Petritis

**Affiliations:** Division of Laboratory Sciences, National Center for Environmental Health, Centers for Disease Control and Prevention, Atlanta, GA 30341, USA; yhx1@cdc.gov (S.L.I.); ogh6@cdc.gov (C.A.P.); nmo1@cdc.gov (R.L.); ijz6@cdc.gov (C.C.)

**Keywords:** newborn screening, high-throughput LC-MS/MS, amino acids, acylcarnitines, dried blood spots

## Abstract

*Background/Objectives*: Mass spectrometry-based newborn screening for small-molecule biomarkers typically employs a rapid first-tier screen that omits chromatographic separations before mass spectrometric analysis, followed, only for a subset of samples and disorders, by a longer, more specific second-tier assay that includes liquid chromatographic separation prior to mass spectrometry. The second-tier screen is used when the primary biomarker lacks sufficient specificity and may result in higher false-positive rates. The throughput and specificity of first-tier newborn screening assays have been relatively stagnant over the past two decades despite significant improvements in mass spectrometry instrumentation. With the continuous expansion of disorders added to the Recommended Uniform Screening Panel in the United States, newborn screening laboratories have a need for higher-throughput assays and improved specificity. *Methods*: We developed and evaluated two first-tier tandem mass spectrometry approaches using a modern dual-needle, dual-loop LC-MS/MS platform: (1) a 30-s flow injection analysis tandem mass spectrometry (FIA-MS/MS) assay and (2) a rapid first-tier liquid chromatography tandem mass spectrometry (LC-MS/MS) assay using a hydrophilic interaction chromatography (HILIC) guard column (1TH). Analytical performance was assessed using dried blood spot quality control and linearity materials, including evaluations of recovery, precision, linearity, and matrix effects. *Results*: The 30-s FIA-MS/MS assay quadrupled the throughput of current 2-min FIA-MS/MS assays used routinely in newborn screening laboratories. The throughput improvement was achievable due to increased scan speeds of the mass spectrometer as well as the dual needle/loop design of the autosampler. In addition, these instrumentation improvements made it possible to employ liquid chromatographic separations prior to MS/MS analysis without sacrificing the approximately 2-min sample-to-sample throughput of conventional FIA-MS/MS workflows. The 1TH LC-MS/MS method separated critical isobaric and isomeric biomarkers, reduced matrix effects, improved specificity and quantification accuracy, and demonstrated acceptable recovery, precision, and linearity for newborn screening applications. *Conclusions*: Recent advances in LC-MS/MS instrumentation can be leveraged to either substantially increase first-tier newborn screening throughput or improve analytical specificity while maintaining current workflow timelines. First-tier LC-MS/MS using a HILIC guard column provides improved specificity that can reduce the need for second-tier testing, thereby improving overall throughput and turnaround time of the newborn screening workflow. These approaches provide flexible solutions for newborn screening laboratories seeking to accommodate expanding screening panels without compromising analytical quality or efficiency.

## 1. Introduction

Mass spectrometry–based first-tier newborn screening (NBS) evaluates over 40 clinically significant biomarkers, providing pre-symptomatic detection of over 30 metabolic disorders [1]. Tandem mass spectrometry–based newborn screening relies on well-established biomarkers for the early detection of serious inherited metabolic disorders, enabling timely treatment before symptom onset and improved clinical outcomes [2]. After birth, a heel prick is performed, blood is collected on a dried blood spot (DBS) card, and the DBS card is sent to an NBS laboratory for analysis. Most biomarkers are screened using a multiplexed flow injection analysis (FIA) tandem mass spectrometry (MS/MS) assay with a throughput of approximately 2 min per sample; however, sub-2 min sample-to-sample analysis is desired [3]. Despite the adoption of FIA-MS/MS in NBS more than two decades ago, there has been little major innovation and/or improvement in analysis throughput [4]. Nevertheless, tandem mass spectrometry continues to expand into new newborn screening applications, including enzymatic assays, steroid profiling, lysosomal disorders, and other emerging targets, increasing the analytical demands placed on existing workflows [5,6]. For instance, many laboratories are still using single-injector, single-loop, nominal-resolving mass spectrometers, with high dwell times required to obtain optimal sensitivity for some biomarkers. In recent years, several new NBS disorders have been added to the Recommended Uniform Screening Panel, so new biomarkers are continually being added to these highly multiplexed assays [7]. The expanding scope of mass spectrometry–based newborn screening now includes biomarkers, enzyme activities, and multiplex assays capable of evaluating disorders traditionally screened using separate analytical platforms [8]. With the demand to multiplex more analytes in a single assay, issues such as isobaric interferences, matrix effects, and throughput create challenges under FIA-MS/MS conditions [9]. Therefore, without hardware advancements, one can anticipate increased false positives and increased workload for follow-up activities as new disorders are continually adopted.

NBS has historically prioritized assay precision, sensitivity, and throughput at the expense of accuracy or specificity. For instance, many current MS-based first-tier assays suffer from inaccuracy due to interferences, use of single-point calibration, use of surrogate internal standards (IS), and the absence of separations prior to analysis (i.e., no chromatography) and calibrators. NBS biomarkers screened by FIA-MS/MS are typically low mass, with isomers and isobars extracted from the DBS or from internal standards [10], which adds complexity to analyses and can yield inconclusive test results [11]. Since DBS extracts undergo no sample clean-up (i.e., no solid phase extraction) and are injected into the MS under FIA conditions, there are matrix effects causing ionization suppression that exceeds 75% for some biomarkers [10]. NBS assays also use single-point calibration for quantitation, as opposed to a linear set of calibrators, which contributes to inaccuracy, especially when comparing data between laboratories [12]. The inaccuracy of NBS assays is further compounded by the use of surrogate internal standards (IS), which do not yield accurate biomarker quantification due to different mass spectrometry responses as a result of structural differences and/or differential ionization due to matrix effects under FIA conditions [13].

The limitations of first-tier NBS are mitigated for some disorders/analytes by employing second-tier screening, which has greatly improved the specificity of MS-based assays [14,15,16,17]. Second-tier screening or chemical derivatization is often required to separate two isobars or isomers that are indistinguishable during first-tier screening by FIA-MS/MS. Screen-positive results from first-tier screening are flagged for reanalysis, where a second DBS punch is prepared and typically analyzed using a method employing liquid chromatographic (LC) separations with sample-to-sample analysis ranging from 3 to 20 min [14,15,16]. In fact, our group recently developed a universal second-tier screening assay capable of screening 19 biomarkers in under 15 min [18]. Second-tier screening increases the time for clinicians to receive results, as it is often completed 1–3 days after first-tier NBS, especially if second-tier screening is not performed in-house and is sent to another laboratory for analysis. The ideal first-tier assay would employ rapid separations to maintain or increase throughput and to separate critical biomarker pairs from interferences, minimizing the number of specimens reflexed to second-tier screening.

Recent advances in ultra-high-pressure liquid chromatography and small-particle-size chromatographic packing materials have begun to reshape the analytical landscape for high-throughput screening assays and have enabled an increasing number of first- and second-tier LC-MS/MS screening applications [5]. For NBS biomarkers such as amino acids, acylcarnitines, succinylacetone, lysophosphatidylcholines and nucleosides, there are several examples of multiplexed assays at 3–7 min; however, these assays focus mainly on a single analyte class or a subset of second-tier screening analytes. For example, Kivilompolo et al. [19] developed a 7 min LC-MS/MS assay for 12 acylcarnitines, LaMarca et al. [20] a 5 min assay for a few organic acids and Shigematsu et al. [21] a 6 min assay for organic acids and homocysteine. Assays that match the FIA-MS/MS (i.e., <2 min) throughput only focused on one analyte and/or disorder [15,22,23]. For some of those assays, although the biomarker of interest may be eluted in <2 min, injection-to-injection throughput was as long as 6 min [22]. Finally, Pickens et al. [13] showed that dozens of first- and second-tier screening NBS biomarkers can be analyzed in <2 min using chip-based electrophoresis coupled to mass spectrometry. Limitations of the assay included long reloading times leading to a total analysis time of about 4 min and inability to analyze biomarkers that were negatively charged in solution, as well as lysophosphatidylcholines (LPCs). To our knowledge, there is no assay that can simultaneously analyze amino acids, acylcarnitines, succinylacetone, lysophosphatidylcholines and nucleosides using fast separations in <2 min.

Depending on the specific newborn screening laboratory, some may have a growing need to multiplex more disorders into mass spectrometry–based workflows. Other labs may screen for fewer disorders but have a need for higher throughput. Therefore, we have developed two different first-tier screening approaches to meet the needs of newborn screening labs: (1) quadrupled throughput of FIA-MS/MS and (2) fast LC-MS/MS. The latest generation of LC-MS/MS instrumentation offers the flexibility to either enhance the throughput of FIA-MS/MS analyses or incorporate chromatographic separations to resolve isomers and isobars before mass spectrometric detection. In this study, we quadruple NBS first-tier FIA-MS/MS analysis throughput by utilizing a dual-needle, dual-loop, fast-scanning LC-MS/MS platform, reducing sample-to-sample analysis from 2 min to <30 s. In addition, we developed a rapid first-tier hydrophilic interaction chromatography (HILIC) guard column (1TH) method on the same LC-MS/MS platform, achieving a sample-to-sample throughput of <2 min. This approach increases result accuracy by separating critical biomarker pairs from interferences, thereby reducing the need for second-tier screening for certain biomarkers. Precision, accuracy, and linearity data were generated for both methods and are presented herein. The LC-MS/MS platform used in this study has a small laboratory footprint with a 16-plate-capacity autosampler, maximizing the analysis capability per square foot of an NBS laboratory. In conclusion, NBS labs could increase throughput, specificity, and accuracy within the same timeframe as the current method by adopting the methods and platform presented in our study.

## 2. Materials and Methods

### 2.1. Dried Blood Spot Samples and Internal Standards

Appropriate safety control measures (including engineering, administrative policy and procedure, and personal protective equipment) were used for all procedures based on a site-specific risk assessment that identified physical, health, and procedural hazards. The Centers for Disease Control and Prevention’s Newborn Screening Quality Assurance Program (NSQAP) quality control (QC) and linearity DBS cards were analyzed in this study [24]. This activity was reviewed by the CDC, was deemed research not involving human subjects, and was conducted in accordance with applicable federal law and CDC policy.

### 2.2. Sample Preparation

Samples were extracted as previously described [25,26]. More details regarding sample extraction, manufacturers, lots of solvents and reagents, and IS used are available in the Supplementary Materials. In brief, a 3.2 mm DBS punch was transferred into a 96-well plate. Next, 100 µL of working IS solution containing 80/20 acetonitrile/water with 0.05% formic acid, 0.015% hydrazine hydrate, and isotopically labeled IS was added to the DBS punch. The plates were heated, sealed and incubated for 45 min at 45 °C. After incubation, DBS extracts were transferred away from the ghost DBS into clean wells of a 96-well plate. The plate was then heat-sealed and placed into an Agilent 1290 Infinity II Multisampler (Agilent Technologies, Santa Clara, CA, USA) autosampler for analysis.

### 2.3. Analysis by Tandem Mass Spectrometry

For the 2 min FIA-MS/MS analyses, sample extracts were analyzed on an Acquity UPLC system coupled to a Waters Xevo TQD tandem mass spectrometer (Waters, Milford, MA, USA). For the 30 s FIA-MS/MS and the 1TH (2 min LC-MS/MS), sample extracts were analyzed on an Agilent 1290 Infinity II LC system coupled to an Agilent Ultivo 6465B LC-MS/MS (Agilent Technologies, Santa Clara, CA, USA). A total of 10 µL was injected for flow injection analyses, 2 µL was injected for LC-MS/MS analyses, and the multisampler was set to 20 °C. Collision energies were optimized using a MassHunter compound optimizer. The parent, product, cone and collision energies, along with dwell times, are specified in Supplementary Table S1.

For the 30 s FIA-MS/MS, a back-pressure regulator was used in place of a traditional LC union to prevent the back pressure from dropping too low and causing the pumps to stall, which can occur with FIA flow profiles. The mobile phase was 50/50/0.02 acetonitrile/water/formic acid, and the flow rate was held at 0.15 mL/min for 0.05 min, increased to 0.50 mL/min from 0.06 to 0.2 min, increased to 2.0 mL/min from 0.21 to 0.28 min, then returned to 0.15 mL/min to prepare for the next injection. For FIA-MS/MS, a dwell time of 10 ms was applied to all MRM transitions. The reduction in total analysis time was achieved through a combination of the dual-needle/dual-loop autosampler architecture, reduced acquisition cycle times, and rapid MRM acquisition enabled by the newer-generation tandem mass spectrometer.

For the 1TH, sample extracts were injected into an Agilent InfinityLab Poroshell HILIC 2.1 × 5 mm 1.9 µm guard column with a fixed column temperature of 45 °C, coupled to the Agilent LC-MS/MS (Agilent Technologies). A short HILIC guard column was selected to provide sufficient retention and separation of critical isobaric analytes while minimizing chromatographic dead time and preserving throughput compatible with first-tier newborn screening. Mobile phase A (MPA) was composed of 90/10 acetonitrile/water containing 0.2% formic acid, 10 mM ammonium formate, and 20 µM oxalic acid. Mobile phase B (MPB) was 100% water, 10 mM ammonium formate, and 20 µM oxalic acid. LC gradient ramps were from 100% MPA to 50% MPB from 0.1 to 0.4 min at 0.75 mL/min, returning to 100% MPA for equilibration. The flow rate was increased to 2.0 mL/min from 0.91 to 1.7 min for faster equilibration between analyses. It should be noted that gradient delay is ~0.5 min at 0.75 mL/min with the quaternary pump (Agilent G7104A), as estimated from instrument pressure traces. Any changes to the system, including switching to a binary pump or changes to the dead volume, will require a modification of the gradient to achieve similar separations. Dynamic MRM (dMRM) was used for acquisition, with the cycle time set to 250 ms. Retention time windows and average dwell times for each analyte under these conditions are included in Supplementary Table S1. Total throughput using this platform with 1TH is approximately 2 min sample-to-sample.

For the comparison of recovery, precision and linearity of the 1TH and 30 s FIA-MS/MS acquired data, QC DBS samples were extracted and analyzed in duplicate on 8 separate days (*n* = 16). The 2 min FIA-MS/MS data were used as a control and consisted of the average of duplicate samples analyzed on 20 separate days (*n* = 40). Linearity material biomarker concentrations are presented in Supplementary Table S2. All biomarker characterization data were analyzed using our 2 min FIA-MS/MS non-derivatized method, except the C3DC and C4OH, which were analyzed using our butyl ester-derivatized 2 min FIA-MS/MS, and the LPCs were analyzed using the Haynes et al. negative mode reverse phase LC-MS/MS method [16].

### 2.4. Data Analysis and Interpretation

Data were acquired and processed on the Agilent Ultivo using MassHunter Workstation LC-MS Data Acquisition V12.1 Update 3 and MassHunter Quantitative Analysis V12.0 (Agilent Technologies, Santa Clara, CA, USA). Peak integration was automatically performed using Agilent MassHunter Quantitative Analysis V12.0 software and reviewed for consistency across injections. Dynamic MRM retention time windows were used to maximize dwell times and maintain sufficient data points across chromatographic peaks while monitoring a large number of transitions. Quantification was performed exactly as in other NBS MS-based assays, using the Relative ISTD option, which determines the analyte concentration from the peak area ratio and internal standard concentration provided. A dilution factor of 32.258 was applied to all specimens to account for the 3.1 μL volume of blood from a 3.2 mm DBS punch extracted in 100 μL IS solution. Quantified data were output into Excel format, and analysis and visualization were performed using R version 4.4.0 [27]. Recovery, precision, and matrix effect calculations were performed using custom R scripts (R version 4.4.0) to ensure consistent processing across analytical runs.

## 3. Results

### 3.1. Comparison of First-Tier Analysis Throughputs

**Figure 1:**
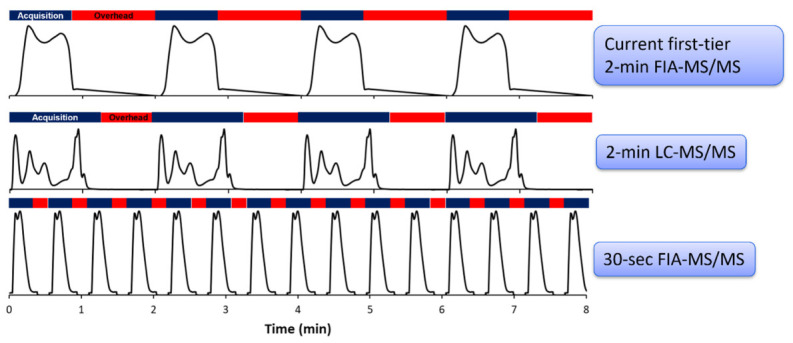
Total ion chromatograms representative of a typical 2 min FIA-MS/MS first-tier analysis, 2 min LC-MS/MS (1TH) analysis, and 30 s FIA-MS/MS analysis. Above each chromatogram, the acquisition is represented by a blue rectangle and the overhead time (needle washing, drawing the next sample, equilibration of LC column, etc.) is represented by a red rectangle.

Sample-to-sample throughput was compared between the 30 s FIA-MS/MS and the 1TH method on the Agilent system, and with the 2 min FIA-MS/MS first-tier method analyzed on a Waters system (Waters, Milford, MA, USA) (Figure 1). Each method used the same preparation described in the Section 2, except the current 2 min FIA-MS/MS sample preparation includes a dry-down and resuspension step. Figure 1 visualizes the total ion chromatogram for each method, demonstrating that the 1TH method achieved similar sample-to-sample throughput to the 2 min FIA-MS/MS, with the added benefit of separations prior to analysis. The 30 s FIA-MS/MS analysis on the Agilent platform quadruples sample-to-sample throughput compared to the 2 min FIA-MS/MS analyzed on the Waters platform.

### 3.2. Separation of Biomarkers on First-Tier HILIC (1TH)

**Figure 2:**
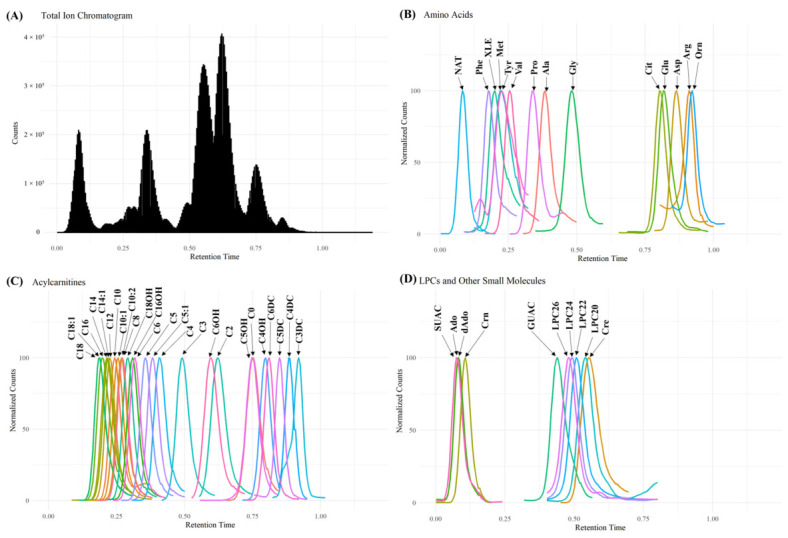
(**A**) Total ion chromatogram, (**B**) XICs of amino acid components, (**C**) XICs of acylcarnitine components, and (**D**) XICs of LPCs and other small molecules from a low QC DBS injection. Each peak in all XICs has been normalized to 100%.

Figure 2 displays the total ion chromatogram (Figure 2A) and normalized overlaid extracted ion chromatograms (XIC) (Figure 2B–D) from a low QC DBS injection. The use of dynamic MRM allows for analysis of over 90 transitions across the 1 min elution window with the narrow LC peaks. Figure 2B displays the XIC for all amino acids (AA) analyzed in our study. Interestingly, the first AA to elute was N-acetyltyrosine (NAT), which is a tyrosine analog added to neonatal parenteral nutrition formulas to boost tyrosine availability. Our group recently published a manuscript demonstrating the utility of NAT as a biomarker of parenteral nutrition in first-tier newborn screening, which can minimize false-positive results due to misannotated DBS cards from neonatal intensive care units [25].

**Figure 3:**
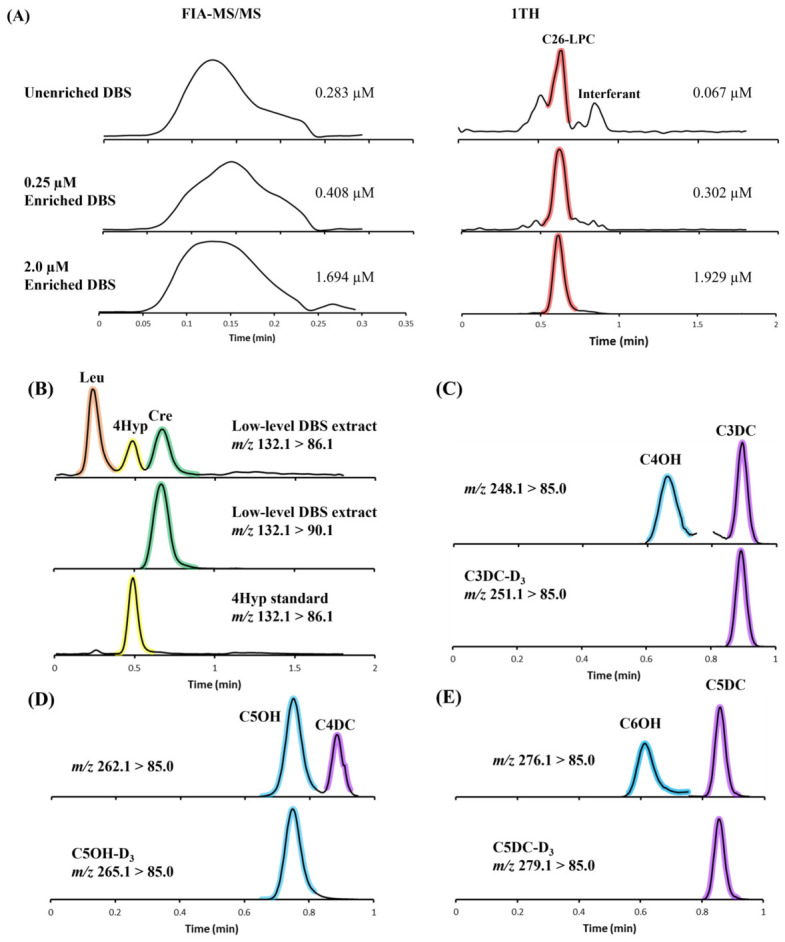
Separation of key isobaric compounds: (**A**) XICs of LPC 26:0 (positive mode *m*/*z* 636.4 > 104) for FIA-MS/MS and 1TH in unenriched DBS, 0.25 and 2 µM enriched DBS. Quantified concentrations are displayed on each XIC for comparison. (**B**) XICs of Leu isobaric interferents, where the Leu MRM transition (*m*/*z* 132.1 > 86.1) has 3 distinct peaks in a DBS extract, the CRE-specific transition (*m*/*z* 132.1 > 90.1) has only one peak in the same extract, and a 4Hyp neat standard injection has the same retention time as the middle peak. (**C**) XICs of isobaric acylcarnitines C3DC and C4OH, where the C3DC-D3 internal standard XIC is provided to confirm that the second peak is C3DC. (**D**) XICs of isobaric acylcarnitines C4DC and C5OH, where the C5OH-D3 internal standard XIC is provided to confirm that the first peak is C5OH. (**E**) XICs of isobaric acylcarnitines C5DC and C6OH, where the C5DC-D3 internal standard XIC is provided to confirm that the second peak is C5DC.

Figure 2C displays the separation of acylcarnitine (AC) isobaric critical pairs that cause inconclusive results during routine NBS of non-derivatized DBS extracts by FIA-MS/MS, such as C3DC and C4OH (*m*/*z* 248.1 > 85), C4DC and C5OH (*m*/*z* 262.1 > 85), and C5DC and C6OH (*m*/*z* 276.1 > 85). The separation of these isobar pairs is highlighted specifically in Figure 3 as well. For instance, C3DC and C4OH are biomarkers of different diseases, so an elevation in one biomarker causes inconclusive results, while elevations in C4DC or C6OH can result in false positives for diseases using C5OH or C5DC as biomarkers, respectively. NBS laboratory FIA-MS/MS analysis of non-derivatized DBS extracts sums the transitions of these isobars (i.e., C3DC + C4OH) and uses the surrogate IS C4-D_3_. Screen-positive specimens for elevated C3DC + C4OH may rely on second-tier screening using LC-MS/MS for differentiation or perform butyl ester derivatization, which shifts the mass of dicarboxy AC away from isobaric hydroxy AC. However, butyl ester derivatization requires hydrochloric acid and ovens for butylation, reducing space in laboratory chemical fume hoods, while the acid corrodes ovens, pipettes, and 96-well plate driers. In fact, butylation ovens, pipettes, and plate driers are often replaced semi-annually depending on the sample volume of an NBS laboratory. Finally, butylation degrades analytes of interest by hydrolyzing esters such as LPCs and ACs. Long-chain ACs such as C16:0 and C18:0 elute very early, while their hydroxylated counterparts, C16:0-OH and C18:0-OH, have longer retention. This suggests the 3-hydroxylated position of the AC chain also interacts with the stationary phase, so separation of additional critical pairs, such as C16:1-OH and C17, would also be achievable on our 1TH method.

Figure 2D displays the separation of numerous other critical biomarkers. LPC 26:0 has an unknown diacyl phospholipid interferent in positive mode FIA-MS/MS analysis (i.e., *m*/*z* 636.4 > 104 and *m*/*z* 636.4 > 184) that causes altered quantification during routine NBS (Figure 3A). Currently in positive mode, this unknown interferent of LPC 26:0 causes some laboratories to reflex up to 3% of daily specimens to second-tier screening for adrenoleukodystrophy, where samples are analyzed using a negative-ion-mode LC-MS/MS method [16]. Negative-ion-mode LPC analysis is often less sensitive than positive-mode analysis; however, the negative-ion-mode product ion is more specific since it is the fatty acyl chain instead of the phosphatidylcholine head group. Our 1TH method separates LPC 26:0 from this unknown interferent, yielding greater accuracy (Figure 3A) while reducing the need for second-tier screening. Figure 3B displays the *m*/*z* 132.1 > 86.1 transition without the use of dynamic MRM windows to visualize the separation of the Leu isomers (XLE) from isobaric compounds 4-hydroxyproline (4Hyp) and creatine (Cre). Similar to dicarboxy and hydroxy acylcarnitine isobars, elevations in 4Hyp and/or Cre can artificially elevate Leu concentrations during FIA-MS/MS analysis, causing false-positive results for maple syrup urine disease. Figure 3C–E demonstrates the additional strengths of using 1TH for first-tier NBS to separate C3DC/C4OH, C4DC/C5OH, and C5DC/C6OH isobaric pairs without derivatization. Further, direct IS are used for C3DC and C4OH, C3DC-D_3_ and C4OH-D_3_, respectively, which increases accuracy by removing differential ionization between biomarkers and surrogate IS.

### 3.3. Recovery, Precision, and Linearity

**Figure 4:**
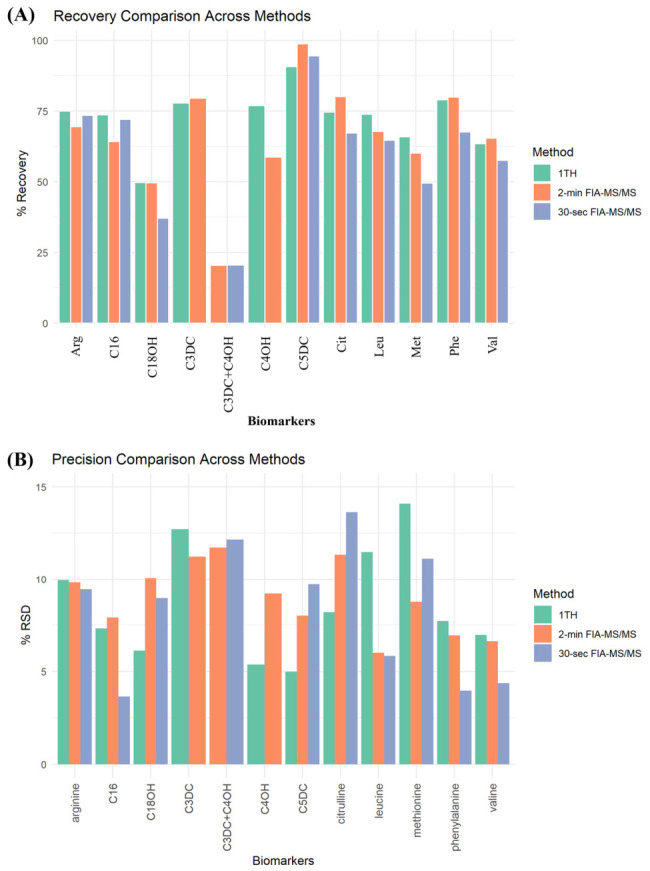
(**A**) Average recovery and (**B**) precision of key biomarkers of interest for 1TH (*n* = 16), 2 min FIA-MS/MS (*n* = 40), and 30 s FIA-MS/MS (*n* = 16).

Biomarker recovery from low QC DBS is presented in Figure 4A. Recovery was calculated by subtracting the base pool experimental concentrations (i.e., non-enriched blood) from low QC biomarker experimental concentrations, then dividing that value by the enrichment concentration of each biomarker in the low QC. Therefore, recovery also accounts for extraction efficiency and metabolism by enzymes present during blood manufacturing. As previously discussed, non-derivatized FIA-MS/MS methods cannot distinguish isobars C3DC and C4OH, so the data are displayed as C3DC + C4OH. Recovery for C3DC and C4OH individually is plotted only for derivatized FIA-MS/MS results and LC-MS/MS, where they are separated. As expected, recovery was similar across the three methods for the select biomarkers displayed in Figure 4A. It is worth noting that C4OH had greater recovery when using the direct IS C4OH-D_3_ in the 1TH method than when using C5DC-D_3_, which is often used as a surrogate in butyl ester-derivatized and second-tier methods. Recovery data for all biomarkers are presented in Supplementary Table S3.

The analytical precision of select biomarkers extracted from low QC DBS is presented in Figure 4B. The standard deviation across the data was divided by the mean, then multiplied by 100; therefore, precision data are presented as the percent residual standard deviation (% RSD). Precision was similar across the three methods; as expected, a precision of ~15% is a common criterion for first-tier NBS assays using MS/MS for analysis. Precision data for all biomarkers are presented in Supplementary Table S4.

Linearity was also evaluated for 1TH and 30 s FIA-MS/MS using the 9-level linearity DBS. These materials were analyzed over six different runs, and the average y-intercept, slope and coefficient of determination (R^2^) are presented in Supplementary Table S5. All analytes exhibited excellent linearity with R^2^ > 0.97 on the 1TH assay. For the 30-s FIA-MS/MS assay, a few analytes had linearity with lower R^2^ which was generally due to the use of surrogate internal standards and lower sensitivity as compared to 1TH.

### 3.4. Matrix Effects

**Figure 5:**
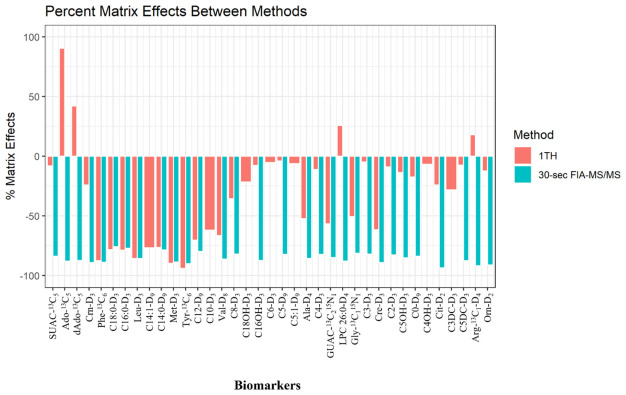
Percent matrix effects for internal standards in DBS extracts as compared to neat solution, where orange bars represent 1TH and teal bars represent 30 s FIA-MS/MS. The *x*-axis is ordered by retention time.

Matrix effects were calculated for 30 s FIA-MS/MS and 1TH methods, using the same data analyzed for recovery and precision, to demonstrate the reduction in matrix effects using separations prior to analysis (Figure 5). The *x*-axis is ordered based on elution time. The *y*-axis displays the percent matrix effects, which were calculated by averaging each IS peak area in neat IS solution (*n* = 64) and low QC DBS extracts (*n* = 64), dividing the difference of the QC and neat IS peak areas by the neat IS peak areas, then multiplying by 100. As expected, significant ion suppression was observed in the 30 s FIA-MS/MS, with an approximately 80% decrease in signal intensity observed in the DBS extract as compared to the neat IS solution for most analytes. By adding LC separations, 1TH achieves improved matrix effects for the majority of analytes. Ion enhancement was observed for a few analytes in 1TH, where the % matrix effects were positive, indicating that the signal intensity of IS in the DBS extract was greater than that observed in the neat solution. The experimental concentrations of these analytes in QC DBS were comparable to the concentrations observed by conventional FIA-MS/MS analysis, supporting that this is a true ion-enhancement observation.

## 4. Discussion

In our current study, we demonstrate the advantages of using a dual-injector, dual-loop, fast-scanning instrument to quadruple first-tier throughput (i.e., 30 s FIA-MS/MS) or improve specificity and accuracy with similar throughput to current first-tier assays, while reducing reflex rates to second-tier assays (i.e., 1TH). Both of these MS methods performed similarly to or better than current methods and technologies used in routine NBS. Other advantages of the LC-MS/MS system used were the small laboratory footprint compared with all current quadrupole-based systems and large autosampler capacity (i.e., 16 plates) integrated into the autosampler. Physical laboratory space constraints are one of the common concerns among NBS laboratories. As more diseases are adopted for routine screening, and as annual births continue rising in many states, there is a limit to the amount of MS instrumentation that can fit into a single room or building. For example, lysosomal disorders recently added to the US Recommended Uniform Screening Panel are mostly screened by MS, which has doubled the number of MS instruments required per NBS laboratory. High-throughput multiplexed assays are critical to the future of biochemical screening in NBS, along with compact, sensitive fast-scanning MS systems with a small laboratory footprint.

While the faster FIA-MS/MS described in the study requires a faster autosampler, the 1TH requires minimal modifications to current instrumentation used in newborn screening labs, only requiring a binary or quaternary LC pump and the installation of a guard column between the autosampler and mass spectrometer inlet. As such, the method is easily translatable across LC-MS/MS instrumentation from various manufacturers. In fact, several NBS laboratories that have inquired about our 1TH method and one US public health laboratory are working toward clinically validating a slightly modified version of the current assay. Advantages of the 1TH assay over FIA approaches include improved specificity, decreased need for second-tier screening, reduced ion suppression, and improved ability to multiplex additional analytes. One of the considerations of 1TH includes the need for actual IS for quantification, as surrogate IS can be chromatographically resolved from the analyte of interest. Also, implementing 1TH in an NBS laboratory introduces the need for peak review, which is not typical in an FIA workflow. Another limitation is the need for additional laboratory technician training. The burden of peak review can be reduced by using a quantification platform with a “review-by-exception” option. Overall, the benefits outweigh the additional considerations of employing separations in first-tier newborn screening, especially with the growing demand to multiplex more analytes in MS-based newborn screening.

Further improvements to 1TH will include multiplexing dozens of additional biomarkers that are relevant to newborn screening, as well as adding polarity switching for analytes that perform better in negative mode, such as organic acids, LPCs, and sulfatides. The extraction conditions will also be further optimized to ensure acceptable recovery of the added biomarkers from dried blood spots. We are also developing an internal standard kit that will contain all the internal standards required in 2–3 vials to facilitate adoption of the method. Finally, we plan to analytically validate the assay and continue to assist laboratories interested in clinical validation. Successful implementation of either approach will depend on laboratory-specific considerations, including instrument compatibility, method validation, workflow integration, and adherence to applicable regulatory and quality assurance requirements.

As additional disorders are considered for inclusion in newborn screening panels, analytical platforms must support increasing multiplexing demands without compromising turnaround times. The approaches presented here provide a pathway for accommodating future biomarkers while maintaining workflows compatible with high-volume public health laboratories.

## 5. Conclusions

This study demonstrates that recent advances in tandem mass spectrometry instrumentation can be leveraged to address two major challenges facing modern newborn screening programs: increasing testing demand and the need for improved analytical specificity. Using a dual-needle, dual-loop LC-MS/MS platform, we developed a 30 s FIA-MS/MS assay that quadruples first-tier screening throughput compared with conventional 2 min FIA-MS/MS methods while maintaining comparable analytical performance. We also developed a rapid first-tier HILIC LC-MS/MS method that incorporates chromatographic separation without sacrificing the throughput expected for routine newborn screening workflows.

The 1TH approach provides several analytical advantages, including separation of clinically important isobaric and isomeric biomarkers, reduced matrix effects, improved quantification through the use of analyte-specific internal standards, and decreased reliance on second-tier testing. These improvements have the potential to reduce false-positive results, shorten turnaround times, decrease the follow-up testing burden, and improve overall screening efficiency. Importantly, the two approaches presented are complementary rather than mutually exclusive. Newborn screening laboratories may choose to adopt either the ultra-high-throughput 30 s FIA-MS/MS assay or the 1TH LC-MS/MS assay based on their screening volumes, disorder panels, existing second-tier testing strategies, available space, staffing resources, instrumentation, and workflow requirements. As newborn screening continues to expand with the addition of new disorders and biomarkers, these flexible high-throughput mass spectrometry approaches provide practical options for enhancing laboratory capacity, specificity, and scalability while maintaining compatibility with the operational needs of public health screening programs.

## Data Availability

The original contributions presented in this study are included in the article/Supplementary Material. Further inquiries can be directed to the corresponding author.

## Supplementary Materials

The following supporting information can be downloaded at: https://www.mdpi.com/article/10.3390/metabo16070443/s1, Table S1: List of metabolite names, *m*/*z*, and internal standards. Surrogates (surr) noted when used in manuscript. Table S2: Linearity material characterized concentrations (µM). Table S3: Percent recovery across the three methods for low QC lot B2215. Table S4: Percent precision across the three methods for low QC lot B2215. Table S5: Linearity results across the two methods (*n* = 3).

## Abbreviations

The following abbreviations are used in this manuscript:

NBS	Newborn screening
DBS	Dried blood spot
FIA	Flow injection analysis
MS/MS	Tandem mass spectrometry
IS	Internal standard(s)
LC	Liquid chromatography
LPC	Lysophosphatidylcholine(s)
HILIC	Hydrophilic interaction chromatography
1TH	Fast first-tier screening using HILIC guard column
NSQAP	The Centers for Disease Control and Prevention’s Newborn Screening Quality Assurance Program
QC	Quality control
MPA	Mobile phase A
MPB	Mobile phase B
dMRM	Dynamic MRM
XIC	Extracted ion chromatogram
AA	Amino acid(s)
NAT	N-acetyltyrosine
%RSD	Percent relative standard deviation
AC	Acylcarnitine(s)
XLE	Leucine isomers
4Hyp	4-hydroxyproline
Cre	Creatine

## References

[1] Wilcken B., Wiley V. (2015). Fifty years of newborn screening. J. Paediatr. Child Health.

[2] Wan Z., Han J., Yang H.S., Luo R.Y., Xie J., Zhai Y., Kong Y., Cao Z. (2026). Tandem mass spectrometry in newborn screening for inherited metabolic diseases: A comprehensive review. Innov. Med..

[3] Pickens C.A., Petritis K. (2020). High resolution mass spectrometry newborn screening applications for quantitative analysis of amino acids and acylcarnitines from dried blood spots. Anal. Chim. Acta.

[4] Chace D.L., Kalas T.A., Naylor E.W. (2003). Use of tandem mass spectrometry for multianalyte screening of dried blood specimens from newborns. Clin. Chem..

[5] Millington D.S. (2024). How mass spectrometry revolutionized newborn screening. J. Mass Spectrom. Adv. Clin. Lab.

[6] Rinaldo P., Tortorelli S., Matern D. (2004). Recent developments and new applications of tandem mass spectrometry in newborn screening. Curr. Opin. Pediatr..

[7] Watson M.S., Mann M.Y., Lloyd-Puryear M.A., Rinaldo P., Howell R., American College of Medical Genetics Newborn Screening Expert Group (2006). Newborn screening: Toward a uniform screening panel and system--executive summary. Pediatrics.

[8] Hong X., Kumar A.B., Ronald Scott C., Gelb M.H. (2018). Multiplex tandem mass spectrometry assay for newborn screening of X-linked adrenoleukodystrophy, biotinidase deficiency, and galactosemia with flexibility to assay other enzyme assays and biomarkers. Mol. Genet. Metab..

[9] Tarini B.A., Christakis D.A., Welch H.G. (2006). State newborn screening in the tandem mass spectrometry era: More tests, more false-positive results. Pediatrics.

[10] Pickens C.A., Courtney E., Isenberg S.L., Cuthbert C., Petritis K. (2023). Multiplexing Homocysteine into First-Tier Newborn Screening Mass Spectrometry Assays Using Selective Thiol Derivatization. Clin. Chem..

[11] De Jesus V.R., Chace D.H., Lim T.H., Mei J.V., Hannon W.H. (2010). Comparison of amino acids and acylcarnitines assay methods used in newborn screening assays by tandem mass spectrometry. Clin. Chim. Acta.

[12] Pickens C.A., Sternberg M., Seeterlin M., De Jesus V.R., Morrissey M., Manning A., Bhakta S., Held P.K., Mei J., Cuthbert C. (2020). Harmonizing Newborn Screening Laboratory Proficiency Test Results Using the CDC NSQAP Reference Materials. Int. J. Neonatal Screen..

[13] Pickens C.A., Isenberg S.L., Cuthbert C., Petritis K. (2021). Combining First and Second-Tier Newborn Screening in a Single Assay Using High-Throughput Chip-Based Capillary Electrophoresis Coupled to High-Resolution Mass Spectrometry. Clin. Chem..

[14] Turgeon C.T., Magera M.J., Cuthbert C.D., Loken P.R., Gavrilov D.K., Tortorelli S., Raymond K.M., Oglesbee D., Rinaldo P., Matern D. (2010). Determination of total homocysteine, methylmalonic acid, and 2-methylcitric acid in dried blood spots by tandem mass spectrometry. Clin. Chem..

[15] Oglesbee D., Sanders K.A., Lacey J.M., Magera M.J., Casetta B., Strauss K.A., Tortorelli S., Rinaldo P., Matern D. (2008). Second-tier test for quantification of alloisoleucine and branched-chain amino acids in dried blood spots to improve newborn screening for maple syrup urine disease (MSUD). Clin. Chem..

[16] Haynes C.A., De Jesus V.R. (2012). Improved analysis of C26:0-lysophosphatidylcholine in dried-blood spots via negative ion mode HPLC-ESI-MS/MS for X-linked adrenoleukodystrophy newborn screening. Clin. Chim. Acta.

[17] de Hora M., Heather N., Webster D., Albert B., Hofman P. (2023). The use of liquid chromatography-tandem mass spectrometry in newborn screening for congenital adrenal hyperplasia: Improvements and future perspectives. Front. Endocrinol..

[18] Kilgore M.B., Platis D., Lim T., Isenberg S., Pickens C.A., Cuthbert C., Petritis K. (2023). Development of a Universal Second-Tier Newborn Screening LC-MS/MS Method for Amino Acids, Lysophosphatidylcholines, and Organic Acids. Anal. Chem..

[19] Kivilompolo M., Ohrnberg L., Oresic M., Hyotylainen T. (2013). Rapid quantitative analysis of carnitine and acylcarnitines by ultra-high performance-hydrophilic interaction liquid chromatography-tandem mass spectrometry. J. Chromatogr. A.

[20] la Marca G., Malvagia S., Pasquini E., Innocenti M., Donati M.A., Zammarchi E. (2007). Rapid 2nd-tier test for measurement of 3-OH-propionic and methylmalonic acids on dried blood spots: Reducing the false-positive rate for propionylcarnitine during expanded newborn screening by liquid chromatography-tandem mass spectrometry. Clin. Chem..

[21] Shigematsu Y., Yuasa M., Ishige N., Nakajima H., Tajima G. (2021). Development of Second-Tier Liquid Chromatography-Tandem Mass Spectrometry Analysis for Expanded Newborn Screening in Japan. Int. J. Neonatal. Screen..

[22] Masse R., Skrinska V., Younes N., Hassan L., Mitri R., Matern D., Rinaldo P., Turgeon C.T. (2017). A Rapid Screening Method for the Measurement of Neonatal Total Homocysteine in Dried Blood Spots by Liquid Chromatography-Tandem Mass Spectrometry. Int. J. Neonatal Screen..

[23] Fingerhut R., Roschinger W., Heck M. (2019). A Rapid and Sensitive UPLC-MS/MS-Method for the Separation and Quantification of Branched-Chain Amino Acids from Dried Blood Samples of Patients with Maple Syrup Urine Disease (MSUD). Int. J. Neonatal Screen..

[24] De Jesus V.R., Mei J.V., Cordovado S.K., Cuthbert C.D. (2015). The Newborn Screening Quality Assurance Program at the Centers for Disease Control and Prevention: Thirty-five Year Experience Assuring Newborn Screening Laboratory Quality. Int. J. Neonatal Screen..

[25] Pickens C.A., Sah S., Chandrappa R., Isenberg S.L., Courtney E.R., Lim T., Chace D.H., Lee R., Cuthbert C., Petritis K. (2024). N-Acetyltyrosine as a Biomarker of Parenteral Nutrition Administration in First-Tier Newborn Screening Assays. Int. J. Neonatal Screen..

[26] Asef C.K., Khaksarfard K.M., De Jesus V.R. (2016). Non-derivatized Assay for the Simultaneous Detection of Amino Acids, Acylcarnitines, Succinylacetone, Creatine, and Guanidinoacetic Acid in Dried Blood Spots by Tandem Mass Spectrometry. Int. J. Neonatal Screen..

[27] R Core Team (2017). R: A Language and Environment for Statistical Computing.

