# Supplementary material for: Improving the Throughput and Specificity for Small-Molecule Analysis During First-Tier Mass Spectrometry–Based Newborn Screening
Source: Metabolites. 2026 Jun 25;16(7):443. doi: 10.3390/metabo16070443 (PMC13413493; doi:10.3390/metabo16070443)
Supplement: Supplementary file 1 [file metabolites-16-00443-s001.zip › metabolites-4344084-supplementary.pdf]

Supplemental Methods

Sample type and preparation

The specimens used in this study included first-tier QC (Lot# A-D2215) and linearity materials (Lot# C20211-C20219) containing amino acids and acylcarnitines. QC DBS cards included a base pool and three enriched versions of the base pool, at varying enrichment levels for each metabolite, and QC production was performed as previously described [1]. First-tier linearity DBS cards included a nine-level set of materials. The DBS cards were prepared by diluting a highly enriched blood pool with non-enriched blood. Additional information regarding pool preparation and concentrations can be found in the Supplemental Table S2 (i.e., linearity) and Quality Control Specimen Certification Data are available on the Center's for Disease Control and Prevention's Newborn Screening Quality Assurance portal.

Chemical, standard, and consumable information

Optima LC/MS Acetonitrile [A955-4], Optima LC/MS Water [W64], Optima LC/MS Methanol [A456-4] (Fisher Scientific, Waltham, MA, USA), LC/MS grade formic acid [A117-50] (Thermo Scientific GmbH, Bremen, Germany), and hydrazine hydrate [225819] (Sigma-Aldrich, Inc, St. Louis, MO, USA) were used in extraction, resuspension, and the mobile phases. Oxalic acid [≥ 99%, 241172-50G] and ammonium formate solution [10 M, 78314-500mL] were used to prepare the mobile phases (Sigma-Aldrich, Inc, St. Louis, MO, USA). All samples were extracted, processed, and resuspended in Corning 3365 96-well round bottom polypropylene plates (VMR International Corp, Suwanee, GA, USA).

Sample extraction

The same extraction protocol was used for preparing first-tier and linearity specimens. Samples were extracted and analyzed as previously described [2], unless otherwise noted. Extraction working IS (WISS) solution was comprised of 80/20 acetonitrile/water, 0.05% formic acid, and 0.015% hydrazine hydrate using the Labeled Amino Acid Standards Set A [NSK-A], Labeled Carnitine Standards Set B [NSK B-1], Labeled Carnitine Standards Supplement to NSK-B [NSK-B-G1-1], creatine [methyl-D_3, DLM-1302], creatinine [N-methyl-D_3, DLM-3653], succinylacetone [3,4,5,6,7-$^{13}C_5$, CLM-6755], guanidinoacetic acid [1,2-$^{13}C_2$, $^{15}N_3$, CNLM-8300], glutamic acid [D_3, DLM-335], glutamine [D_5, DLM-1826], proline [$^{13}C_5$, CLM-2260], aspartic acid [D_3 99%, DLM-546-0], C10 [D_3 DLM-9067-0], C6 [D_3, DLM-9276], C3DC [D_3, DLM-11049], C5:1 [D_9, DLM-12325], C14:1 [D_9, DLM-12326], LPC 26:0 [D_4, DLM-10501-0], adenosine [$^{13}C_5$, CLM-3678], deoxyadenosine [$^{13}C_5$, CLM-4579], and N-acetyltyrosine [$^{13}C_6$, CLM-11320] (Cambridge Isotope Laboratories Inc., Andover, MA, USA). C6OH [54253] and C4OH [D_3 79301] were purchased from MilliporeSigma (Burlington, MA, USA). C6DC [870873] was purchased from Avanti Polar Lipids, Inc. (Alabaster, AL, USA). C6OH and C6DC were spiked into DBS extract to demonstrate separation capabilities on the first-tier HILIC (1TH) method.

The source parameters for both FIA and 1TH methods were as follows: capillary voltage 4000 V, nozzle voltage 2000 V, gas flow 10.0 L/min, nebulizer 60.0 psi, sheath gas flow 11.0 L/min, gas temperature 300 °C, and sheath gas temperature 350 °C.

Supplemental Table S1: List of metabolite name, *m/z*, and internal standards. Surrogates (surr) noted when used in manuscript.

Metabolite (Biomarker Short Name)	Precursor > Product (m/z)	RT ± window (min)	Fragmentor (V)	CE (V)	Average dwell (ms)	Internal Standard
Succinylacetone-hydrazone (SUAC)	155.1 > 137.1	0.09 ± 0.1	86	8	8.77	SUAC-$^{13}C_5$
SUAC-$^{13}C_5$-hydrazone (SUAC-$^{13}C_5$)	160.1 > 142.1	0.09 ± 0.1	86	8	8.77	
Creatinine (Crn)	114.1 > 44.1	0.1 ± 0.1	114	16	10.97	Crn-D_3
Creatinine-$^2H_3$ (Crn-D_3)	117.1 > 47.1	0.1 ± 0.1	114	16	10.97	
N-acetyltyrosine (NAT)	224.1 > 136.1	0.1 ± 0.1	86	12	10.97	NAT-$^{13}C_6$
N-acetyltyrosine-$^{13}C_6$ (NAT-$^{13}C_6$)	230.1 > 142.1	0.1 ± 0.1	86	12	10.97	
Adenosine (**Ado**)	268.1 > 136	0.1 ± 0.2	80	16	10.99	Ado-$^{13}C_5$
Adenosine-$^{13}C_5$ (Ado-$^{13}C_5$**)**	273.1 > 136	0.1 ± 0.2	80	16	10.99	
Deoxyadenosine (dAdo)	252.1 > 136	0.1 ± 0.2	80	12	10.99	dAdo-$^{13}C_5$
Deoxyadenosine-$^{13}C_5$ (dAdo-$^{13}C_5$**)**	257.1 > 136	0.1 ± 0.2	80	12	10.99	
Phenylalanine (Phe)	166.1 > 120.1	0.18 ± 0.1	86	12	6.65	Phe-$^{13}C_6$
Phenylalanine-$^{13}C_6$ (Phe-$^{13}C_6$)	172.1 > 126.1	0.18 ± 0.1	86	12	6.65	
Leucine (Leu)	132.1 > 86.1	0.19 ± 0.1	86	8	6.33	Leu-D_3
Leucine-$^2H_3$ (Leu-D_3)	135.1 > 89.1	0.19 ± 0.1	86	8	6.33	
Palmitoylcarnitine (C16:0)	400.4 > 85	0.2 ± 0.1	162	32	6.11	C16:0-D_3
Palmitoylcarnitine-$^2H_3$ (C16:0-D_3)	403.4 > 85	0.2 ± 0.1	162	32	6.11	
Tetradecanoylcarnitine (C14:0)	372.3 > 85	0.21 ± 0.1	162	28	5.91	C14:0-D_9
Tetradecenoylcarnitine (C14:1)	370.3 > 85	0.21 ± 0.1	162	28	5.91	
Tetradecenoylcarnitine (C14:1-D_9)	379.1 > 85	0.21 ± 0.1	162	28	5.91	C14:0-D_9 (surr)<br>C14:1-D_9
Tetradecanoylcarnitine-$^2H_9$ (C14:0-D_9)	381.4 > 85	0.21 ± 0.1	162	28	5.91	C14:1-D_9
Stearoylcarnitine (C18:0)	428.4 > 85	0.21 ± 0.1	162	32	5.91	C18:0-D_3
Oleoylcarnitine (C18:1)	426.4 > 85	0.21 ± 0.1	162	32	5.91	C18:0-D_3 (surr)
Stearoylcarnitine-$^2H_3$ (C18:0-D_3)	431.4 > 85	0.21 ± 0.1	162	32	5.91	
Methionine (Met)	150.1 > 104	0.22 ± 0.1	86	8	5.94	Met-D_3
Methionine-$^2H_3$ (Met-D_3)	153.1 > 107	0.22 ± 0.1	86	8	5.94	
Tyrosine (Tyr)	182.1 > 136.1	0.22 ± 0.1	86	12	5.94	Tyr-$^{13}C_6$
Tyrosine-$^{13}C_6$ (TYR-$^{13}C_6$)	188.1 > 142.1	0.22 ± 0.1	86	12	5.94	
Dodecanoylcarnitine (C12)	344.3 > 85	0.23 ± 0.15	162	28	6.9	C12-D_9
Dodecanoylcarnitine-$^2H_9$ (C12-D_9)	353.3 > 85	0.23 ± 0.15	162	28	6.9	
Decanoylcarnitine (C10:0)	316.2 > 85	0.26 ± 0.1	124	24	6.33	C8-D_3 (surr)<br>C10:0-D_3
Decenoylcarnitine (C10:1)	314.2 > 85	0.26 ± 0.1	124	24	6.33	C8-D_3 (surr)<br>C10:0-D_3
Decadienoylcarnitine (C10:2)	312.2 > 85	0.26 ± 0.1	124	24	6.33	C8-D_3 (surr)<br>C10:0-D_3
Decanoylcarnitine-$^2H_3$ (C10:0-D_3)	319.1 > 85	0.26 ± 0.1	124	24	6.33	
Valine (Val)	118.1 > 72.1	0.26 ± 0.1	86	8	6.33	Val-D_8
Valine-$^2H_8$ (Val-D_8)	126.1 > 80.1	0.26 ± 0.1	86	8	6.33	
Octanoylcarnitine (C8)	288.2 > 85	0.28 ± 0.1	124	24	6.81	C8-D_3

Octanoylcarnitine-$^{2}H_3$ (C8-D_3)	291.2 > 85	0.28 ± 0.1	124	24	6.81	
Hydroxystearoylcarnitine (C18OH)	444.4 > 85	0.3 ± 0.1	86	32	7.44	C18:0-D_3 (surr)<br>C18OH-D_3
Hydroxystearoylcarnitine-$^{2}H_3$ (C18OH-D_3)	447.4 > 85	0.3 ± 0.1	86	32	7.44	
Hydroxyhexadecanoylcarnitine (C16OH)	416.4 > 85	0.32 ± 0.1	86	32	7.54	C16OH-D_3
Hydroxyhexadecanoylcarnitine-$^{2}H_3$ (C16OH-D_3)	419.4 > 85	0.32 ± 0.1	86	32	7.54	
Hexanoylcarnitine (C6)	260.2 > 85	0.33 ± 0.1	105	20	7.6	C5-D_9 (surr)<br>C6-D_3
Hexanoylcarnitine-$^{2}H_3$ (C6-D_3)	263.1 > 85	0.33 ± 0.1	105	20	7.6	
Proline (Pro)	116 > 70	0.35 ± 0.1	75	15	7.81	Pro-$^{13}C_5$
Proline-$^{13}C_5$ (Pro-$^{13}C_5$)	121.1 > 74	0.35 ± 0.1	75	15	7.81	
Isovalerylcarnitine (C5:0)	246.2 > 85	0.36 ± 0.1	124	24	7.95	C5-D_9
Isovalerylcarnitine-$^{2}H_9$ (C5-D_9)	255.2 > 85	0.36 ± 0.1	124	24	7.95	
Alanine (Ala)	90 > 44	0.4 ± 0.1	72	8	8.63	Ala-D_4
Alanine (Ala-D_4)	94 > 48	0.4 ± 0.1	72	8	8.63	
Butyrylcarnitine (C4)	232.2 > 85	0.4 ± 0.1	92	24	8.63	C4-D_3
Butyrylcarnitine-$^{2}H_3$ (C4-D_3)	235.2 > 85	0.4 ± 0.1	92	24	8.63	
Tiglylcarnitine (C5:1)	244.2 > 85	0.4 ± 0.1	100	20	8.63	C5-D_9 (surr)<br>C5:1-D_9
Tiglylcarnitine-$^{2}H_9$ (C5:1-D_9)	253.1 > 85	0.4 ± 0.1	100	20	8.63	
Guanidinoacetic acid (GUAC)	118.1 > 76	0.44 ± 0.125	83	8	9.08	GUAC-$^{13}C_2{}^{15}N_1$
Guanidinoacetic acid-$^{13}C_2{}^{15}N$ (GUAC-$^{13}C_2{}^{15}N$)	121.1 > 79	0.44 ± 0.125	83	8	9.08	
Glycine (Gly)	76 > 30	0.47 ± 0.125	72	4	10.08	Gly-$^{13}C^{15}N$
Glycine (Gly-$^{13}C^{15}N$)	78 > 32	0.47 ± 0.125	72	4	10.08	
Propionylcarnitine (C3)	218.1 > 85	0.51 ± 0.1	86	20	9.92	C3-D_3
Propionylcarnitine-$^{2}H_3$ (C3-D_3)	221.2 > 85	0.51 ± 0.1	86	20	9.92	
Creatine (Cre)	132.1 > 90	0.57 ± 0.125	71	12	10.99	Cre-D_3
Creatine-$^{2}H_3$ (Cre-D_3)	135.1 > 93	0.57 ± 0.125	71	12	10.99	
Glutamine (Gln)	147.1 > 84	0.58 ± 0.1	75	15	10.9	Gln-D_5
Glutamine-$^{2}H_5$ (Gln-D_5)	152.1 > 89	0.58 ± 0.1	75	15	10.9	
LPC 20:0	552.4 > 104.1	0.6 ± 0.2	175	28	10.12	LPC 26:0-D_4
LPC 22:0	580.4 > 104.1	0.6 ± 0.2	175	32	10.12	LPC 26:0-D_4
LPC 24:0	608.4 > 104.1	0.6 ± 0.2	175	32	10.12	LPC 26:0-D_4
LPC 26:0	636.4 > 104.1	0.6 ± 0.2	198	32	10.12	LPC 26:0-D_4
LPC 26:0-$^{2}H_4$ (LPC 26:0-D_4)	640.4 > 104.1	0.6 ± 0.2	198	32	10.12	
3-Hydroxyhexanoylcarnitine (C6OH)	276.1 > 85	0.62 ± 0.1	124	24	10.85	
Acetylcarnitine (C2)	204.1 > 85	0.65 ± 0.1	86	20	10.61	C2-D_3
Acetylcarnitine-$^{2}H_3$ (C2-D_3)	207.1 > 85	0.65 ± 0.1	86	20	10.61	
Free Carnitine (C0)	162.1 > 103	0.75 ± 0.1	124	16	9.75	C0-D_9
Free Carnitine-$^{2}H_9$ (C0-D_9)	171.2 > 103	0.75 ± 0.1	124	16	9.75	
Hydroxyisovalerylcarnitine (C5OH)	262.2 > 85	0.75 ± 0.2	124	24	10.16	C5OH-D_3

Hydroxyisovalerylcarnitine-$^2H_3$ (C5OH-D_3)	265.2 > 85	0.75 ± 0.2	124	24	10.16	
Citrulline (Cit)	176.1 > 113	0.8 ± 0.15	86	12	9.81	Cit-D_2
Citrulline-$^2H_2$ (Cit-D_2)	178.1 > 115	0.8 ± 0.15	86	12	9.81	
Hydroxybutyrylcarnitine (C4OH)	248.1 > 85	0.81 ± 0.1	120	20	9.5	C4-D_3 (surr) C4OH-D_3
Malonylcarnitine-$^2H_3$ (C4OH-D_3)	251.1 > 85	0.81 ± 0.1	120	20	9.5	
Adipoylcarnitine (C6DC)	290.1 > 85	0.82 ± 0.1	86	24	9.53	
Glutamic acid (Glu)	148.1 > 84	0.83 ± 0.15	75	15	10.36	Glu-D_3
Glutamic acid-$^2H_3$ (Glu-D_3)	151.1 > 87	0.83 ± 0.15	75	15	10.36	
Glutarylcarnitine (C5DC)	276.1 > 85	0.85 ± 0.1	86	24	9.71	C5DC-D_3
Glutarylcarnitine-$^2H_3$ (C5DC-D_3)	279.1 > 85	0.85 ± 0.1	86	24	9.71	
Aspartic acid (Asp)	134.1 > 88	0.88 ± 0.1	75	5	10.61	Asp-D_3
Aspartic acid-$^2H_3$ (Asp-D_3)	137.1 > 91	0.88 ± 0.1	75	5	10.61	
Arginine (Arg)	175.1 > 70.1	0.9 ± 0.1	86	24	11.89	Arg-$^{13}C_1$-D_4
Arginine-$^{13}C_1{}^2H_4$ (Arg-$^{13}C_1D_4$)	180.1 > 75.1	0.9 ± 0.1	86	24	11.89	
Lysine (Lys)	147.1 > 84	0.9 ± 0.2	80	16	18.75	
Malonylcarnitine (C3DC)	248.1 > 85	0.92 ± 0.1	120	20	13.53	C4-D_3 (surr) C3DC-D_3
Malonylcarnitine-$^2H_3$ (C3DC-D_3)	251.1 > 85	0.92 ± 0.1	120	20	13.53	
Ornithine (Orn)	133.1 > 70	0.94 ± 0.1	86	16	16.06	Orn-D_2
Ornithine-$^2H_2$ (Orn-D_2)	135.1 > 72	0.94 ± 0.1	86	16	16.06	

Supplemental Table S2. Linearity material characterized concentrations (µM).

Biomarker	20231	20232	20233	20234	20235	20236	20237	20238	20239
Ado	0.28	0.41	0.54	0.77	1.48	2.64	4.95	7.12	9.48
Ala	192.50	204.30	219.75	236.41	287.39	381.98	560.57	732.61	919.09
Arg	8.75	12.59	15.93	22.72	44.59	83.57	161.82	242.13	325.86
C0	18.89	22.88	27.06	34.74	57.12	94.83	167.66	239.00	313.52
C2	10.54	14.22	17.83	24.72	45.33	79.38	143.93	207.66	272.55
C3	1.15	1.61	2.07	2.95	5.67	10.07	18.37	26.53	34.94
‡C3DC	0.03	0.09	0.15	0.28	0.65	1.27	2.51	3.78	5.06
C3DC+C4OH	0.10	0.16	0.22	0.34	0.69	1.27	2.41	3.54	4.73
C4	0.10	0.20	0.30	0.49	1.07	2.02	3.82	5.64	7.52
‡C4OH	0.14	0.22	0.30	0.44	0.90	1.62	2.97	4.32	5.68
C5:1	0.02	0.13	0.23	0.44	1.08	2.13	4.06	5.94	7.89
C5	0.07	0.27	0.46	0.86	2.03	3.93	7.46	10.84	14.25
C5DC	0.04	0.15	0.25	0.46	1.08	2.11	4.08	6.10	8.07
C5OH	0.65	0.81	0.97	1.25	2.13	3.57	6.30	9.06	11.86
C6	0.02	0.12	0.22	0.42	1.01	1.97	3.75	5.49	7.25
C8	0.03	0.53	1.00	1.99	4.90	9.73	18.75	27.38	36.45
C10	0.08	0.37	0.65	1.22	2.88	5.63	10.71	15.67	20.82
C10:1	0.01	0.13	0.23	0.45	1.12	2.17	4.09	5.96	7.85
C10:2	0.01	0.15	0.28	0.54	1.34	2.62	4.97	7.38	9.82
C12	0.02	0.19	0.36	0.70	1.73	3.45	6.78	10.14	13.62
C14:1	0.02	0.16	0.29	0.56	1.38	2.78	5.43	8.23	11.09
C14	0.06	0.26	0.45	0.85	2.03	4.01	7.85	11.74	15.83
C16	0.80	1.20	1.61	2.37	4.66	8.44	15.88	23.36	31.12
C16OH	0.01	0.17	0.31	0.62	1.52	3.03	5.97	8.96	12.09
C18:1	0.91	1.07	1.24	1.49	2.28	3.66	6.21	8.81	11.40
C18	0.51	0.73	0.95	1.37	2.60	4.68	8.81	12.99	17.38
C18OH	0.01	0.07	0.13	0.26	0.63	1.26	2.48	3.71	4.99
Cit	13.06	27.85	41.47	69.87	151.25	291.63	555.41	804.63	1064.69
Cre	226.40	237.23	249.39	258.56	291.89	356.30	474.43	593.09	722.24
Crn	19.25	24.64	30.50	41.59	74.85	129.19	235.08	340.14	449.24
dAdo	0.10	0.19	0.28	0.46	1.00	1.88	3.61	5.31	7.05
Gly	203.63	218.92	237.34	260.98	332.28	463.30	712.76	955.71	1219.64
GUAC	1.20	1.58	1.97	2.74	5.11	8.96	16.01	23.68	30.89
Leu	46.22	67.02	86.94	126.89	245.04	441.46	820.33	1196.66	1587.52
†LPC 24:0	0.05	0.13	0.22	0.39	0.90	1.75	3.53	5.26	6.98
†LPC 26:0	0.02	0.11	0.20	0.39	0.93	1.84	3.73	5.56	7.42
Met	5.54	9.92	14.45	23.51	50.36	96.12	184.55	273.93	366.07
Orn	61.48	69.56	74.02	83.35	105.93	152.20	236.48	317.41	405.43
Phe	16.02	37.13	56.77	98.27	220.28	425.79	820.68	1211.43	1611.36
SUAC	0.28	0.72	1.12	1.98	4.48	8.93	19.22	28.56	40.01
Tyr	18.60	32.63	46.09	75.03	157.73	299.33	574.08	847.40	1131.56
Val	43.79	53.04	63.01	80.73	133.99	225.18	397.83	569.24	748.45

Data are presented as the characterized value from linearity material certification reports for lots 20231-20239 and were analyzed as non-derivatized sample extracts by FIA-MS/MS unless otherwise denoted. Concentrations are presented as µM.

‡ denotes data reported from these biomarkers are from butyl ester derivatized FIA-MS/MS analysis, since these two isobars cannot be distinguished by non-derivatized FIA-MS/MS analysis.

† denotes data reported from these biomarkers were acquired from non-derivatized sample extracts analyzed by LC-MS/MS in negative mode.

Supplemental Table S3. Percent recovery across the three methods for low QC lot B2215.

Biomarkers	1TH (n=16)	2-min FIA-MS/MS (n=40)	30-sec FIA-MS/MS (n-16)
Ala	80.47	71.62	68.44
Ado	83.14	75.09	83.04
Arg	74.93	69.46	73.45
C0	110.21	97.13	101.08
C2	107.74	81.18	90.78
C3	85.07	73.6	74.38
C3DC	77.82	79.52‡	
C3DC+C4OH		20.41	20.51
C4	64.56	58.98	62.46
C4OH	76.89	58.71‡	
**C5**	76.74	76.53	75.67
C5:1	64.53	52.5	55.71
C5DC	90.66	98.7	94.47
C5OH	65.49	56.78	64.37
C6	69.48	62.47	64.22
C8	89.74	84.6	88.91
C10	63.07	82.62	73.86
C12	70.44	62.01	70.94
C14	87.15	86.9	85.4
C14:1	81.67	40.52	44.1
C16	73.63	64.18	72.04
C16OH	87.41	84.41	85.45
C18	73.14	66.17	72.88
C18OH	49.64	49.53	37.08
Cit	74.57	80.12	67.2
Cre	94.46	62.79	62.9
Crn	64.77	78.72	57.45
dAdo	92.74	95.54	211.22
Gly	49.95	62.55	39.5
GUAC	81.29	73.49	56.06
Leu	73.82	67.73	64.68
LPC 24:0	95.12	92.58†	87.22
LPC 26:0	83.5	102.39†	83.81
Met	65.84	60.14	49.46
Orn	82.55	60.3	77.07
Phe	78.97	79.92	67.61
SUAC	33.02	40	31.87
Tyr	99.37	75.08	63.39
Val	63.36	65.43	57.51

‡ denotes data reported from these biomarkers are from butyl ester FIA-MS/MS analysis, since these two isobars cannot be distinguished by non-derivatized FIA-MS/MS analysis.

† denotes data reported from these biomarkers were acquired from non-derivatized sample extracts analyzed by LC-MS/MS in negative mode.

Supplemental Table S4. Percent precision across the three methods for low QC lot B2215.

Biomarkers	1TH (n=16)	2-min FIA-MS/MS (n=40)	30-sec FIA-MS/MS (n-16)
Ala	8.89	7.02	7.71
Ado	3.91	11.54	16.98
Arg	9.97	9.81	9.43
C0	6.99	5.18	6.22
C2	9.42	8.47	6.68
C3	6.46	8.11	4.56
C3DC	12.71	11.24‡	
C3DC+C4OH		11.73	12.16
C4	4.3	9.91	4.35
C4OH	5.38	9.21‡	
C5	3.18	7.21	4.11
C5:1	5.45	9.01	8.51
C5DC	4.99	8.01	9.71
C5OH	3.46	7.79	4.17
C6	3.87	8.02	4.27
C8	4.44	9.1	3.69
C10	5.74	9.41	4.82
C12	6.88	9.21	3.09
C14	10.76	8.93	4.61
C14:1	13.76	10.58	5.01
C16	7.32	7.91	3.65
C16OH	5.55	8.83	7.44
C18	5.52	7.76	4.45
C18OH	6.13	10.07	8.96
Cit	8.2	11.34	13.64
Cre	3.25	7.42	8.43
Crn	4.41	6.87	5.59
dAdo	4.12	14.20	12.71
Gly	11.85	6.29	17.35
GUAC	7.43	12.85	21.09
Leu	11.47	6.01	5.83
LPC 24:0	8.59	12.19†	23.15
LPC 26:0	10.88	8.62†	25.58
Met	14.1	8.75	11.12
Orn	10.22	10.31	9.55
Phe	7.72	6.94	3.97
SUAC	8.01	14.76	10.11
Tyr	16.34	7.18	7.4
Val	6.98	6.62	4.37

‡ denotes data reported from these biomarkers are from butyl ester FIA-MS/MS analysis, since these two isobars cannot be distinguished by non-derivatized FIA-MS/MS analysis.

† denotes data reported from these biomarkers were acquired from non-derivatized sample extracts analyzed by LC-MS/MS in negative mode.

Supplemental Table S5. Linearity results across the two methods (*n=3*).

	1TH			*30-sec FIA-MS/MS*		
**Biomarkers**	**y-intercept**	**slope**	**R^2**	**y-intercept**	**slope**	**R^2**
Ado	0.19	1.09	0.99	0.55	1.21	0.99
Ala	159.02	0.62	0.99	224.46	0.65	0.98
Arg	9.37	0.55	0.99	15.10	1.14	0.99
C0	23.14	0.58	0.99	24.06	0.58	0.99
C2	12.51	1.20	0.99	15.18	1.08	0.99
C3	1.31	0.72	0.99	1.66	0.68	0.99
C3DC	-0.02	0.53	0.99			
C3DC+C4OH				0.23	0.30	0.99
C4	0.12	0.74	0.99	0.18	0.73	0.99
C4OH	0.17	0.72	0.99			
C5	0.04	0.80	0.99	0.34	0.59	0.99
C5:1	-0.15	2.38	0.99	0.26	0.87	0.98
C5DC	0.03	0.89	0.99	0.22	0.79	0.99
C5OH	0.42	0.58	0.99	0.66	0.52	0.99
C6	0.01	0.85	0.99	0.14	0.66	0.99
C8	0.20	0.86	0.99	0.49	0.88	0.99
C10	0.08	0.70	0.99	0.25	0.80	0.99
C10:1	0.07	0.38	0.99	0.09	0.38	0.99
C10:2	0.06	0.51	0.99	0.13	0.46	0.99
C12	0.11	0.68	0.99	0.12	0.68	0.99
C14	0.12	0.78	0.99	0.18	0.75	0.98
C14:1	0.02	0.72	0.99	-0.34	2.25	0.96
C16	0.99	0.71	0.97	0.98	0.63	0.98
C16OH	0.03	1.29	0.99	-0.05	0.63	0.95
C18	0.55	0.84	0.99	0.46	0.77	0.99
C18:1	1.10	0.58	0.99	0.54	0.95	0.82
C18OH	0.01	0.55	0.99	-0.01	0.33	0.96
Cit	9.95	0.52	0.99	37.54	0.73	0.99
CRE	194.32	0.58	0.99	204.20	0.46	0.95
CRN	12.98	0.83	0.99	21.76	0.76	0.99
dAdo	0.04	0.85	0.99	1.32	1.16	0.96
Gly	99.32	0.36	0.99	147.65	0.30	0.92
GUAC	0.46	0.69	0.99	3.89	0.56	0.98
Leu	45.23	0.71	0.99	59.47	0.66	0.99
LPC 24:0	0.03	0.73	0.99			
LPC 26:0	-0.01	0.56	0.99			
Met	1.48	0.75	0.98	9.61	0.72	0.99
Orn	93.73	0.80	0.97	81.13	0.79	0.99
Phe	16.52	0.84	0.99	29.78	0.72	0.99
SUAC	0.20	0.34	0.99	2.51	0.37	0.99
Tyr	1.24	0.89	0.99	43.36	0.70	0.99
Val	39.22	0.66	0.99	54.54	0.61	0.99

References

1. De Jesus, V.R., et al., *The Newborn Screening Quality Assurance Program at the Centers for Disease Control and Prevention: Thirty-five Year Experience Assuring Newborn Screening Laboratory Quality.* Int J Neonatal Screen, 2015. **1**(1): p. 13-26.
2. Asef, C.K., K.M. Khaksarfard, and V.R. De Jesus, *Non-derivatized Assay for the Simultaneous Detection of Amino Acids, Acylcarnitines, Succinylacetone, Creatine, and Guanidinoacetic Acid in Dried Blood Spots by Tandem Mass Spectrometry.* Int J Neonatal Screen, 2016. **2**(4).
